# Supplementary material for: Synchrony between brain age and internalising and externalising symptoms across adolescence
Source: Commun Biol. 2026 Jul 21;9:1509. doi: 10.1038/s42003-026-10722-x (PMC13755446; doi:10.1038/s42003-026-10722-x)
Supplement: Supplementary file 1 [file 42003_2026_10722_MOESM1_ESM.pdf]

nature portfolio

Peer Review File

Synchrony between brain age and internalising and externalising symptoms across adolescence

Corresponding Author: Dr Dani Beck

This file contains all reviewer reports in order by version, followed by all author rebuttals in order by version.

Version 0:

Reviewer comments:

Reviewer #1

(Remarks to the Author)
The manuscript by Beck et al., titled "Synchrony between brain age and internalising and externalising symptoms across adolescence," examines deviations from normative brain maturation (operationalized as the brain-age gap) and their longitudinal associations with internalizing and externalizing symptoms in males and females over seven years. This is a timely and important question, and the manuscript makes a genuine contribution to our understanding of (non-)normative brain development and its relationship to mental health vulnerability in adolescence. The longitudinal design and the different imaging modalities examined show a particular strength of the manuscript. We believe this work would be a valuable addition to the literature, and we suggest the following major and minor revisions:

Major comments:

1. It is a bit unclear how the reported Ns for each modality were arrived at. The authors mention QC and data cleaning but do not report how this impacted the sample selection. Further, it may be helpful to foreshadow the train and test allocation when describing the overall ends to make the numbers more intuitive. Finally, how were sociodemographic variables considered when forming the train and test sets?

2. The use of bivariate latent growth curve (BLGC) models is a principled choice for the research question, and the authors are encouraged to make this case more explicitly clear in the manuscript. In particular, it would be helpful to explain why this framework was preferred over more conventional alternatives (such as generalized additive mixed models (GAMMs), which can capture non-linear trajectories without requiring pre-specified functional forms) and what the BLGC approach uniquely offers. Alongside this, a gentler explanation of what the growth factors (intercept, slope, quadratic) represent, and the intuition for how to think about them would considerably improve accessibility for readers less familiar with this structural equation modelling approach—particularly since many results focus couplings between quadratic components.

3. A primary concern with the approach and findings is that the authors do not consider socioeconomic status (SES). SES is a robust predictor of both neurobiological and mental health outcomes as well as a modulator of developmental trajectories. It would be very important to know if the BAG approach is simply indexing SES-related differences. This could also be a consideration in the creation of the train and test groups. Another important variable to consider would be pubertal timing. The authors' allude to the role of puberty in the discussion but do not examine it empirically. Both of these variables warrant careful consideration and inclusion in the analytic approach. Accounting for these variables would substantially strengthen confidence in the reported BAG–symptom associations and the interpretability. Both variables are available within the ABCD dataset.

4. Several of the figures would benefit from a revision. Figure 1 is currently missing a color legend within the figure itself. In Figure 4, the y-axis label could be made clearer, the x-axis label is underneath the legend which makes it hard to find, the panel ordering is somewhat difficult to follow, and it is not immediately apparent which associations reach statistical significance. It is also worth noting that cross-referencing an earlier figure within a figure legend can be difficult for readers who are not viewing both simultaneously. More broadly, the figure legends represent an opportunity to shortly summarize the key results more directly, expanding them so that each figure is interpretable as a standalone element would improve the overall readability of the paper.

Minor comments:
1. It is not clear why there are more batches than sites in the combat harmonization procedure. More details on this would be helpful.

2. The brain age gap procedures look to be appropriate and avoid the pitfalls identified in Butler et al. (2021, Human Brain Mapping). The authors should include that important brain age gap paper in the citations and point out that their approach aligns with the paper's recommendations.

3. It would be helpful if the manuscript consistently clarified whether reported p-values are corrected or uncorrected. Line 429 specifies that FDR-corrected values are used, but everywhere else only p is reported without qualification.

4. The introduction could be strengthened by providing a more thorough grounding in sex differences in internalizing and externalizing psychopathology, including which groups are more commonly affected and at which developmental stages. A clearer definition of what internalizing and externalizing symptoms entail would also be good and would provide a stronger foundation for the sex-related findings reported later in the paper.

5. The authors should revise the concluding statement saying that "vulnerability arises not from absolute levels of neurobiological features, but from asynchronies in their timing and pace of change." The analyses, as we understand them, identify coupling between deviations from "normative" developmental trajectories (essentially residuals after accounting for age) and how those deviations co-fluctuate (rather that rate of change in brain per-se). There is also not a direct assessment of lead-lag asynchrony. It seems a more grounded statement would be that "vulnerability arises not from absolute levels of neurobiological features, but from deviations from canonical developmental profiles."

Reviewer #2

(Remarks to the Author)
Beck et al. examine whether trajectories of brain age gap (BAG) are coupled with internalizing and externalizing symptom trajectories across adolescence, using four models (T1, dMRI, rs-fMRI, multimodal) and bivariate latent growth curve (BLGC) models in the ABCD study. The main takeaway is that nonlinear changes in BAG are positively coupled with symptom worsening, particularly for internalizing symptoms in females. The longitudinal design is a clear step forward from prior cross-sectional work, but I have a number of concerns that need to be addressed prior to publication.

1. MacSweeney et al. (2025, Biological Psychiatry: CNNI), from the same group with overlapping authors, already looked at longitudinal multimodal brain age-internalizing associations in ABCD. The current paper adds more waves, externalizing symptoms, and BLGC models – which are meaningful extensions – but calling this "the first study to apply multimodal brain age modelling within a longitudinal framework" overstates this novelty. The introduction should be more upfront about how this builds on MacSweeney et al.

2. The paper states that family-aware splitting was appropriately applied to ensure siblings weren't split across training and test sets. However, the CV folds used for hyperparameter tuning and the 80/20 bias-correction split within the training half do not appear to group by family ID. The authors should clarify whether family structure was actually respected at all stages, and if not, assess the impact.

3. The authors use BPM rather than full CBCL, which ABCD also collects. I assume this is because the BPM was administered at more waves, giving better temporal resolution to fit the symptom trajectories, and is also self-reported. But this rationale is never stated. The fact that the study uses a self report is actually a considerable strength of the study.

4. Eight BLGC models are fitted (4 modalities x 2 symptom domains), each producing multiple coupling parameters. While FDR correction is applied to the sex-difference tests, as far as I can tell, no correction is applied to the primary BAG-symptom coupling tests themselves. The authors should apply FDR correction across these tests as well.

5. The BLGC models don't include any covariates (e.g., SES, race/ethnicity, pubertal status, site), all of which have been shown to impact internalizing/externalizing symptoms. A sensitivity analysis with key covariates would strengthen the manuscript.

6. The effect sizes are mostly small, or small-to-medium at best. Some discussion on what this means in practical or clinical terms would be helpful.

7. In the GitHub repository, there's no code at all for the BLGC models, the model selection procedure, or sex-difference tests. Code is also needed here for reproducibility.

8. The repository also references ABCD release 5.1 with two waves, but the paper uses release 6.0 with four waves. The discrepancy should be clarified.

Reviewer #3

(Remarks to the Author)

This manuscript leverages multiple time points from the ABCD study to examine how trajectories in brain age gap (BAG) in different imaging modalities (structure, function, DWI, multimodal) relate to internalizing and externalizing symptomology. This paper is generally well-written and theoretically motivated, and fills a clear gap in the literature around longitudinal studies mapping BAG and transdiagnostic symptomology in youth. However, my enthusiasm for this paper is dampened by the lack of specificity in which neural pathways are implicated in these developmental processes. I provide suggestions for improving the manuscript below.

1) Overall, it's unclear what can be gleaned about neural pathways linked to symptomology when hundreds of neural measures are included. In other words, there is a lack of specificity in the findings that make it difficult to interpret beyond the idea that brain maturation rates relate to symptomology, which is interesting but not at all mechanistic. This is especially evident in the discussion, which does not provide any interpretation of the development of specific neural networks, regions, or other neural processes supporting adolescent behavior and psychopathology.

2) Could the authors clarify why they did not examine additional polynomials beyond quadratic effects, given that there is not a lot of existing literature examining longitudinal BAG, and because they have enough time points to model more complex functions in the data?

3) I understand that the authors have provided variable names for transparency, but for figures like S4, they could provide what the acronym means or label them in a way that is easier for readers to digest.

4) They report test performance in Table 1, but the paper would be more transparent if it also reported how the training and validation set performed (e.g., MAE, RMSE, R^2, age bias, and residual age correlations).

5) The authors seem to frame neurodevelopment and psychopathology as separate processes in a few places in the intro and discussion (e.g., first sentence of the discussion). The authors may consider rephrasing to acknowledge that neurodevelopment underlies the development of psychopathology, not that they happen to occur in tandem.

6) For adolescent neurodevelopment, chronological age alone is often insufficient. Given the authors' claims about developmental timing and sex differences in the discussion, pubertal status, pubertal timing, or hormone related measures should be considered as covariates, moderators, or at least acknowledged as a stronger limitation. They note this in discussion, but this feels central to their argument/interpretation.

7) The authors acknowledge the limitations of the BPM in their discussion, but they should more clearly state in the limitations that only symptoms from the prior week were assessed.

Version 1:

Reviewer comments:

Reviewer #1

(Remarks to the Author)
I appreciate the authors' thoughtful and responsive responses. The inclusion of the SES-related analyses adds considerable rigor to the approach and adds confidence in the interpretation of the results. The acknowledgement of limitations to incorporating pubertal timing in this approach is also helpful. The authors have addressed my concerns and have greatly improved the manuscript.

Reviewer #2

(Remarks to the Author)
I thank the authors for taking the time to address my concerns. I have no further concerns.

Reviewer #3

(Remarks to the Author)
Thank you to the authors for thoroughly addressing my comments. I have no other concerns about the manuscript, and it is much improved this round!

Reviewer #4

(Remarks to the Author)
I co-reviewed this manuscript with one of the reviewers who provided the listed reports. This is part of the Communications Biology initiative to facilitate training in peer review and to provide appropriate recognition for Early Career Researchers who co-review manuscripts.

Open Access This Peer Review File is licensed under a Creative Commons Attribution 4.0 International License, which permits use, sharing, adaptation, distribution and reproduction in any medium or format, as long as you give appropriate credit to the original author(s) and the source, provide a link to the Creative Commons license, and indicate if changes were made.
In cases where reviewers are anonymous, credit should be given to 'Anonymous Referee' and the source.
The images or other third party material in this Peer Review File are included in the article's Creative Commons license, unless indicated otherwise in a credit line to the material. If material is not included in the article's Creative Commons license and your intended use is not permitted by statutory regulation or exceeds the permitted use, you will need to obtain permission directly from the copyright holder.
To view a copy of this license, visit https://creativecommons.org/licenses/by/4.0/

Reviewers' comments:

Reviewer #1 (Remarks to the Author):

The manuscript by Beck et al., titled "Synchrony between brain age and internalising and externalising symptoms across adolescence," examines deviations from normative brain maturation (operationalized as the brain-age gap) and their longitudinal associations with internalizing and externalizing symptoms in males and females over seven years. This is a timely and important question, and the manuscript makes a genuine contribution to our understanding of (non-)normative brain development and its relationship to mental health vulnerability in adolescence. The longitudinal design and the different imaging modalities examined show a particular strength of the manuscript. We believe this work would be a valuable addition to the literature, and we suggest the following major and minor revisions:

Major comments:

1. It is a bit unclear how the reported Ns for each modality were arrived at. The authors mention QC and data cleaning but do not report how this impacted the sample selection. Further, it may be helpful to foreshadow the train and test allocation when describing the overall ends to make the numbers more intuitive. Finally, how were sociodemographic variables considered when forming the train and test sets?

Response: We thank Reviewer 1 for these suggestions. We have addressed all three sub-points in the revised manuscript and supplementary material.

Regarding sample derivation: we have added a detailed description of the QC exclusion cascade per modality to SI Section 3, reporting the number of observations before and after each QC step at each timepoint. Briefly, T1-weighted data were reduced from 30,276 to 29,594 observations, DTI from 29,165 to 25,949, and rs-fMRI from 29,526 to 26,061, following exclusion of participants with excessive head motion or poor data quality based on the ABCD team's recommendations (abcd_imgincl01). The paragraph now ends by directing readers to SI Tables 1 and 2 for the full demographic breakdown of the resulting training and test sets.

Regarding foreshadowing the train/test split: we have added a brief framing sentence to the Demographic information and data quality assurance section of the main manuscript, clarifying upfront that the reported sample sizes refer to the hold-out test set, with the full sample having been divided 50/50 into training and test sets prior to analysis. This should make the reported Ns more immediately interpretable for readers.

Regarding sociodemographic variables in the split: the partition was performed using a family-aware group shuffle split, ensuring that all observations from a given individual and their siblings were assigned to the same set. Age and sex distributions were additionally verified to be comparable across the training and test sets, as can be confirmed in SI Tables 1 and 2. While SES and other sociodemographic variables were not used as explicit stratification variables, the large sample size and random assignment of families to partitions beyond the family and age constraints makes systematic sociodemographic imbalance between the training and test sets unlikely. More directly, we note that the SES sensitivity carried out during this revision – in which a composite baseline SES measure was included as a time-invariant covariate in all BLGC models – provides empirical reassurance that the reported BAG–symptom associations are robust to socioeconomic variation, regardless of any potential distributional differences between partitions.

The revised supplementary material has been updated (SI Section 3) with the following addition:

"For MRI data, quality check procedures followed the standard protocol described in Hagler et al. (2019)[9]. Briefly, participants with excessive head motion or poor data quality were excluded from the curated data release by the ABCD team. Additional quality assurance was carried out following extraction of data using the recommendations for data cleaning provided by the ABCD team (using data structure abcd_imgincl01), where recommendation criteria for include/exclude is marked by 1/0 respectively, and removal of data marked 0 was carried out. Following this procedure, T1-weighted data was reduced from 30,276 observations (TP1: 11,755; TP2: 8,092; TP3: 6,343; TP4: 4,086) to 29,594 (TP1: 11,324; TP2: 7,897; TP3: 6,296; TP4: 4,077). DTI data was reduced from 29,165 observations (TP1: 11,130; TP2: 7,842; TP3: 6,224; TP4: 3,969) to 25,949 (TP1: 10,237; TP2: 6,838; TP3: 5,459; TP4: 3,415), and rs-fMRI data from 29,526 observations (TP1: 11,269; TP2: 7,924; TP3: 6,274; TP4: 4,059) to 26,061 (TP1: 9,394; TP2: 6,940; TP3: 5,896; TP4: 3,831). *The dataset for the multi-modal brain age model was formed by merging the aforementioned data sets. The sample was then split into brain age training and test sets, the demographics of which are documented in SI Tables 1 and 2."*

The revised main manuscript has been updated (lines 196–198) with the following framing sentence added at the start of the Demographic information and data quality assurance section:
"For each modality, the full sample was divided into training (50%) and hold-out test (50%) sets prior to analysis, with all observations from a given individual assigned to the same set; the sample sizes reported below refer to the hold-out test set used in all subsequent analyses."

2. The use of bivariate latent growth curve (BLGC) models is a principled choice for the research question, and the authors are encouraged to make this case more explicitly clear in the manuscript. In particular, it would be helpful to explain why this framework was preferred over more conventional alternatives (such as generalized additive mixed models (GAMMs), which can capture non-linear trajectories without requiring pre-specified functional forms) and what the BLGC approach uniquely offers. Alongside this, a gentler explanation of what the growth factors (intercept, slope, quadratic) represent, and the intuition for how to think about them would considerably improve accessibility for readers less familiar with this structural equation modelling approach—particularly since many results focus couplings between quadratic components.

Response: We thank Reviewer 1 for this constructive suggestion. We agree that a clearer justification for the BLGC framework and a more accessible explanation of the growth factors strengthens the manuscript, and we have revised the Statistical analysis section accordingly.

Regarding the choice of BLGC over GAMMs: both frameworks can accommodate nonlinear change, but they address fundamentally different inferential goals. GAMMs are well suited to estimating flexible population-average trajectories without requiring a pre-specified functional form. However, they do not naturally decompose individual differences in development into interpretable latent components, nor do they provide a unified framework for estimating the covariance structure between two parallel developmental processes at the person level. The central question of this paper – whether individuals who show accelerating deviations in brain maturation also show accelerating symptom trajectories – requires exactly this: person-level growth components for both BAG and symptoms estimated simultaneously, with their cross-process covariances as primary parameters of interest. The BLGC framework provides this directly. We also note that flexible nonlinear specifications sharing the spirit of GAMMs – specifically latent basis slopes, which freely estimate the shape of change from the data – were considered and tested during model development. These produced inadmissible solutions across modalities due to insufficient information for freely estimated nonlinear forms given the four-wave MRI schedule (see SI Section 5), further supporting the use of a structured quadratic parameterisation.

The revised manuscript has been updated (lines 311–319): *"The BLGC framework was selected because it simultaneously decomposes individual differences in two parallel developmental processes into interpretable latent growth components and permits direct estimation of their covariance structure. In this framework, the intercept captures each individual's characteristic level at the*

centred timepoint, the linear slope captures the overall pace and direction of change across adolescence, and the quadratic component captures whether that rate of change itself accelerates or decelerates – a distinction particularly relevant given the nonlinear developmental patterns characteristic of both brain maturation and psychopathology in adolescence."

3. A primary concern with the approach and findings is that the authors do not consider socioeconomic status (SES). SES is a robust predictor of both neurobiological and mental health outcomes as well as a modulator of developmental trajectories. It would be very important to know if the BAG approach is simply indexing SES-related differences. This could also be a consideration in the creation of the train and test groups. Another important variable to consider would be pubertal timing. The authors' allude to the role of puberty in the discussion but do not examine it empirically. Both of these variables warrant careful consideration and inclusion in the analytic approach. Accounting for these variables would substantially strengthen confidence in the reported BAG–symptom associations and the interpretability. Both variables are available within the ABCD dataset.

Response: We thank Reviewer 1 for raising these important points, and we agree that SES and pubertal timing are theoretically meaningful variables that deserve careful consideration in the context of this work. We address each in turn.

Regarding the primary aim of the study, we wish to first clarify the motivating research question. The BLGC framework was selected specifically to characterise the degree to which individual differences in brain maturational trajectories and symptom trajectories co-develop across adolescence – that is, to ask whether the developmental tempo of brain maturation and psychopathology are synchronised at the individual level, above and beyond the normative age-related trends already accounted for by the BAG approach. The goal was not to model all potential sources of variance in brain development or mental health, nor to adjudicate between competing explanatory mechanisms such as genetic, socioeconomic, or hormonal factors. These are important and complementary questions, but they are distinct from the developmental coupling question we address here.

That said, we fully appreciate that SES is one of the most robust predictors of both brain development and mental health outcomes, and that without sensitivity testing it is reasonable to ask whether the reported associations are driven by SES-related gradients in brain maturation and symptom trajectories. We have therefore conducted a sensitivity analysis in which a composite baseline measure of socioeconomic status – combining parental and partner income and educational attainment – was included as a time-invariant covariate regressed onto all latent growth factors in each BLGC model. The results were highly consistent with the primary model estimates across all modalities and symptom domains, with negligible attenuation of the quadratic–quadratic coupling estimates that form the core of our findings. This provides direct evidence that the reported BAG–symptom associations are not simply indexing SES-related differences in developmental trajectories. The SES-adjusted coupling estimates are reported in full in SI Table 11, and the sensitivity analysis is described in the methodology and results sections of the revised manuscript.

Lines 339-343: "*To assess robustness of primary coupling estimates to socioeconomic confounding, a sensitivity analysis was conducted including a composite baseline measure of socioeconomic status (SES; combining parental and partner income and educational attainment) as a time-invariant covariate regressed onto all latent growth factors; results are reported in Section 3.2.4 and SI Table 11.*"

Lines 534-541 now read: "*Sensitivity analyses: To assess the robustness of primary coupling estimates to socioeconomic confounding, a sensitivity analysis was conducted in which a composite measure of socioeconomic status (SES; combining parental and partner income and educational attainment at baseline) was included as a time-invariant covariate regressed onto all latent growth factors in each BLGC model. Coupling estimates from the SES-adjusted models were highly consistent with primary model estimates across all modalities and symptom domains, supporting the robustness of the reported associations (SI Table 11).*"

Regarding SES and the train/test split, the primary 50/50 train/test split was performed using a family-aware group shuffle procedure that simultaneously balanced longitudinal versus cross-sectional participation across the two halves, with age and sex distributions verified to be comparable across sets as shown in SI Tables 1 and 2. While SES was not used as an explicit stratification variable, the large sample size and random assignment of families beyond age and sex constraints makes systematic SES imbalance between partitions unlikely. More importantly, the SES sensitivity analysis carried out during this revision – in which a composite baseline SES measure was included as a time-invariant covariate regressed onto all latent growth factors in each BLGC model – provides direct empirical reassurance that the core BAG–symptom associations are robust to socioeconomic variation, and the near-identical validation and test set performance metrics across all four modalities (reported in SI Section 3) confirm that the brain age models generalise well to the held-out test set.

Regarding pubertal timing, we agree this is theoretically important and have given it careful consideration. However, comprehensive longitudinal modelling of pubertal effects within the BLGC framework is not straightforward in the present dataset. PDS data in ABCD release 6.0 has incomplete longitudinal coverage across the observation window, meaning that a time-varying pubertal covariate could not be included without substantially reducing the analytic sample or introducing additional missingness into an already sparse four-wave MRI schedule. A baseline-only PDS covariate would not capture the developmental tempo of pubertal change that is most theoretically relevant to the coupling question and would risk conflating pubertal status at a single timepoint with the intercept of the developmental trajectory. Furthermore, the primary goal of the BLGC framework was to characterise coordinated developmental change between brain maturation and symptom trajectories, rather than to decompose sources of individual differences in those trajectories. Sex was modelled explicitly as a grouping variable throughout, providing partial accounting for the systematic sex differences in pubertal timing that are most relevant here. We have acknowledged pubertal timing as a limitation in the revised manuscript. The revised manuscript includes the following addition to the Strengths and Limitations section:

Lines 696-712: "Seventh, although pubertal timing was not included as a covariate in the present analyses, we acknowledge this as a meaningful limitation given the centrality of puberty to adolescent brain maturation and the emergence of internalising psychopathology. The inclusion of sex as a grouping variable provides partial accounting for systematic sex differences in pubertal tempo, and the SES sensitivity analysis offers reassurance that findings are not attributable to a major sociodemographic confound. Nonetheless, pubertal timing, hormonal change, and their interactions with brain maturational trajectories represent important unmeasured factors, and future work incorporating longitudinal pubertal assessments within a developmental coupling framework would be a valuable extension of the present findings."

4. Several of the figures would benefit from a revision. Figure 1 is currently missing a color legend within the figure itself. In Figure 4, the y-axis label could be made clearer, the x-axis label is underneath the legend which makes it hard to find, the panel ordering is somewhat difficult to follow, and it is not immediately apparent which associations reach statistical significance. It is also worth noting that cross-referencing an earlier figure within a figure legend can be difficult for readers who are not viewing both simultaneously. More broadly, the figure legends represent an opportunity to shortly summarize the key results more directly, expanding them so that each figure is interpretable as a standalone element would improve the overall readability of the paper.

Response: We thank Reviewer 1 for these detailed suggestions. Regarding the missing colour legend in Figure 1, we considered adding a formal colour legend but ultimately retained the current approach for the following reason. The figure has a structurally complex first wave, where a BPM density distribution and a separate MRI age distribution are overlaid in the same panel using two distinct fills. A two-category legend (e.g., 'BPM only' vs 'MRI + BPM') would not accurately represent this overlap

and could mislead readers into thinking each colour maps cleanly to a single data source across all waves. The figure caption provides detailed information about the colour coding and the distinction between BPM-only and MRI-aligned waves, and we believe this is a more accurate and informative approach than a legend that would oversimplify the structure of the figure. We have reviewed the caption to ensure it is sufficiently clear and self-contained for readers viewing the figure in isolation.

For Figure 4, we have updated the y-axis label from "Model-implied symptom level" to "Predicted symptom score" for clarity, and moved the sex legend from the bottom to the right-hand side of the figure, which resolves the overlap with the x-axis label and gives both labels clear visual prominence. Regarding panel ordering, the panels deliberately mirror the modality and outcome sequence of Figure 3 to facilitate cross-referencing between the two figures; we have made this explicit in the revised caption so readers can orient themselves without needing to view both simultaneously.

Regarding statistical significance, Figure 4 is designed to illustrate the shape of predicted symptom trajectories at high and low values of the BAG growth factors and is not intended as an inferential display – significance of the underlying couplings is reported in full in the Results section and supplementary material. Following the reviewer's suggestion that figure legends should allow standalone interpretation, we have also revised the Figure 4 caption to remove cross-references to other figures and tables, ensuring it is self-contained.

The revised Figure 4 caption reads:
"Figure 4. Predicted symptom trajectories conditional on brain age gap. The figure visualises the BLGC coupling effects by plotting predicted internalising and externalising symptom trajectories at low (−1 SD; solid lines) versus high (+1 SD; dotted lines) values of the corresponding BAG growth component. Panels are ordered by modality (T1, dMRI, rs-fMRI, multimodal) with internalising preceding externalising within each modality. Trajectories are shown separately for females (gold) and males (burgundy) where sex-stratified models were estimated; for dMRI externalising, trajectories reflect pooled-sex estimates (grey), consistent with the equality-constrained model selected for that modality. For each modality × outcome coupling combination, the plotted trajectories represent expected symptom development obtained by conditioning the relevant symptom growth factor (intercept, linear slope, or quadratic component) on the corresponding BAG factor, while holding all remaining latent growth factors fixed at their estimated means. Because symptom trajectories are parameterised with quadratic growth functions in the final models, conditional trajectories may appear curved even when visualising intercept–intercept or slope–slope couplings."

Minor comments:
1. It is not clear why there are more batches than sites in the combat harmonization procedure. More details on this would be helpful.

Response: We thank Reviewer 1 for flagging this. The ABCD Study collected data at 21 sites using 34 scanners, as some sites operated multiple scanner models or underwent scanner upgrades during the course of data collection. LongComBat was applied at the scanner level rather than the site level, as scanner hardware is the primary source of systematic MRI variation. We have clarified this in SI Section 2.

The revised supplementary material has been updated (SI Section 2):
"Note that the number of batches (34) exceeds the number of recruitment sites (21) because some sites operated more than one scanner model or underwent scanner upgrades during data collection. LongComBat was therefore applied at the scanner level, as this represents the primary unit of technical variability in multi-site MRI data."

2. The brain age gap procedures look to be appropriate and avoid the pitfalls identified in Butler et al. (2021, Human Brain Mapping). The authors should include that important brain age gap paper in the citations and point out that their approach aligns with the paper’s recommendations.

Response: We thank Reviewer 1 for this suggestion. Butler et al. (2021, Human Brain Mapping) is an important methodological reference for BAG estimation and we have added it to the citations in addition to a methodological paper with specific recommendations for neurodevelopmental samples. We have also added a sentence to the Brain age prediction section explicitly noting that our approach – including the application of a statistical age-bias correction and evaluation of model performance in a held-out test set – is consistent with the recommendations outlined in that paper.

The revised manuscript has been updated (lines 273–276) with the following sentence added after the description of the BAG correction procedure:
"This procedure, including age-bias correction of raw BAG values and evaluation of model performance in an independent hold-out sample, is consistent with methodological recommendations for brain age gap estimation (Butler et al. 2021; Whitmore & Beck 2025)."

3. It would be helpful if the manuscript consistently clarified whether reported p-values are corrected or uncorrected. Line 429 specifies that FDR-corrected values are used, but everywhere else only p is reported without qualification.

Response: We thank Reviewer 1 for this observation and agree that consistent p-value labelling is important for transparency. In response to this comment and Reviewer 2's related concern about FDR correction of primary coupling tests, we have now applied Benjamini–Hochberg FDR correction to all BAG–symptom coupling tests and updated the manuscript accordingly. P-values reported for coupling estimates throughout the Results section now reflect FDR-corrected values and are labelled as *p FDR* consistently. A sentence has been added to the Statistical analysis section of the Methods and the main Results explicitly stating that the p-values are BH FDR-corrected. The Figure 3 caption has also been updated to clarify that asterisks reflect FDR-corrected significance thresholds.

4. The introduction could be strengthened by providing a more thorough grounding in sex differences in internalizing and externalizing psychopathology, including which groups are more commonly affected and at which developmental stages. A clearer definition of what internalizing and externalizing symptoms entail would also be good and would provide a stronger foundation for the sex-related findings reported later in the paper.

Response: We thank Reviewer 1 for this suggestion. We agree that a clearer grounding in the definitions and sex-differentiated developmental patterns of internalising and externalising symptoms would strengthen the introduction and provide a firmer foundation for the sex-related findings reported later in the paper.

The revised manuscript has been updated (lines 83–89), replacing that sentence with the following:
"Adolescence is also a period of rising sex differences in internalising and externalising psychopathology: internalising symptoms – including anxiety, depression, and somatic complaints – show a marked female predominance that emerges during early adolescence and peaks in mid-adolescence, whereas externalising symptoms – including attention problems, aggression, and rule-breaking behaviour – tend to be more prevalent in males, particularly for conduct and attention-related difficulties[22–24]. These diverging patterns coincide with sex differences in the timing and tempo of normative brain maturation[2,25]. Together, these findings suggest that deviations from normative brain maturation trajectories – whether accelerated or delayed – may be centrally implicated in the emergence of youth psychopathology[26]."

5. The authors should revise the concluding statement saying that "vulnerability arises not from absolute levels of neurobiological features, but from asynchronies in their timing and pace of change." The analyses, as we understand them, identify coupling between deviations from "normative" developmental trajectories (essentially residuals after accounting for age) and how those deviations co-fluctuate (rather that rate of change in brain per-se). There is also not a direct assessment of lead-lag asynchrony. It seems a more grounded statement would be that "vulnerability arises not from absolute levels of neurobiological features, but from deviations from canonical developmental

profiles.”

Response: We thank Reviewer 1 for this careful reading. The reviewer is correct that the term "asynchronies" overstates what the analyses directly assess. The BLGC models capture covariation between deviations from age-expected brain maturation and symptom trajectories – they do not directly test lead-lag relationships or asynchrony between neural systems. We have revised the concluding statement accordingly.

The revised manuscript has been updated (lines 719–730): *"The results support a broader developmental view in which vulnerability arises not from absolute levels of neurobiological features, but from deviations from canonical developmental profiles – patterns in which an individual's brain maturational trajectory diverges from age-expected norms in tandem with worsening symptom trajectories, offering a framework for understanding why adolescence is a sensitive period for emerging mental health problems."*

Reviewer #2 (Remarks to the Author):

Beck et al. examine whether trajectories of brain age gap (BAG) are coupled with internalizing and externalizing symptom trajectories across adolescence, using four models (T1, dMRI, rs-fMRI, multimodal) and bivariate latent growth curve (BLGC) models in the ABCD study. The main takeaway is that nonlinear changes in BAG are positively coupled with symptom worsening, particularly for internalizing symptoms in females. The longitudinal design is a clear step forward from prior cross-sectional work, but I have a number of concerns that need to be addressed prior to publication.

1. MacSweeney et al. (2025, Biological Psychiatry: CNNI), from the same group with overlapping authors, already looked at longitudinal multimodal brain age-internalizing associations in ABCD. The current paper adds more waves, externalizing symptoms, and BLGC models – which are meaningful extensions – but calling this “the first study to apply multimodal brain age modelling within a longitudinal framework” overstates this novelty. The introduction should be more upfront about how this builds on MacSweeney et al.

Response: We thank Reviewer 2 for this important point. The reviewer is correct that MacSweeney et al. (2025), from our group, previously examined longitudinal multimodal brain age associations with internalising symptoms in ABCD using two waves, and that our claim of novelty as stated oversteps what the present paper contributes. We have revised the relevant sentence to explicitly acknowledge this prior work and reframe our contribution accurately.

The revised manuscript has been updated (lines 571–576): *"Building on prior longitudinal work examining multimodal brain age and internalising symptoms across two waves in ABCD57, the present study extends this framework to four MRI waves, ten symptom assessment waves, both internalising and externalising symptom domains, and bivariate latent growth curve modelling that explicitly characterises coupled developmental trajectories and their growth factor components.”*

2. The paper states that family-aware splitting was appropriately applied to ensure siblings weren't split across training and test sets. However, the CV folds used for hyperparameter tuning and the 80/20 bias-correction split within the training half do not appear to group by family ID. The authors should clarify whether family structure was actually respected at all stages, and if not, assess the impact.

Response: We thank Reviewer 2 for this careful and important methodological point. We are happy to clarify exactly which stages of the pipeline were and were not family-aware. The primary 50/50 train/test split was fully family-aware. Families were identified using the ABCD family ID variable (ab_g_stc_design_id_fam) and entire family units were assigned to either the training or test set

together, while simultaneously balancing the proportion of longitudinal versus cross-sectional observations across the two halves. No family was ever split across the training and test sets at this stage. Within the training set, however, family structure was not explicitly preserved at two subsequent stages: the 80/20 internal train/validation split used for bias correction, and the 5-fold cross-validation used for hyperparameter tuning. Both of these steps split by participant ID rather than family ID, meaning that in principle, siblings within the training set could have appeared in different folds or partitions. The practical impact of this is likely modest for two reasons. First, both the CV tuning and the 80/20 bias correction split operate entirely within the training set – the hold-out test set remains completely family-clean throughout and is never contaminated. Second, hyperparameter tuning via cross-validation is a relatively robust procedure; slight optimism in hyperparameter selection due to sibling non-independence within folds would be expected to have negligible effects on final model performance in the independent test set, where family separation was strictly maintained. Nonetheless, we acknowledge this as a limitation and have added a sentence to the Strengths and Limitations section of the manuscript.

The revised manuscript has been updated (lines 685–689) with the following sentence added to the Strengths and Limitations section: *"Regarding the brain age prediction pipeline, although the primary train/test split was fully family-aware, family structure was not preserved within the internal cross-validation folds used for hyperparameter tuning or the 80/20 bias-correction split; while the hold-out test set remained family-clean throughout, this represents a minor limitation that may have introduced slight optimism in hyperparameter selection."*

3. The authors use BPM rather than full CBCL, which ABCD also collects. I assume this is because the BPM was administered at more waves, giving better temporal resolution to fit the symptom trajectories, and is also self-reported. But this rationale is never stated. The fact that the study uses a self report is actually a considerable strength of the study.

Response: We thank Reviewer 2 for this observation. The reviewer is correct that the rationale for selecting the BPM over the CBCL was never stated. We have added a sentence to the Brief Problem Monitor measures section of the Methods explaining this choice.

The revised manuscript has been updated (lines 283–286) with the following sentence added after the initial description of the BPM: *"The self-report BPM was selected over the parent-reported CBCL, which is also collected in ABCD, because it was administered at up to ten longitudinal waves in ABCD release 6.0 compared with approximately four waves for the CBCL, providing substantially greater temporal resolution for modelling symptom trajectories within the BLGC framework."*

4. Eight BLGC models are fitted (4 modalities x 2 symptom domains), each producing multiple coupling parameters. While FDR correction is applied to the sex-difference tests, as far as I can tell, no correction is applied to the primary BAG-symptom coupling tests themselves. The authors should apply FDR correction across these tests as well.

Response: We thank Reviewer 2 for this important methodological point and agree that FDR correction should be applied to the primary coupling tests for consistency with the sex-difference testing procedure. We have applied Benjamini–Hochberg FDR correction across all primary BAG–symptom coupling tests. The core findings are robust to this correction. All quadratic–quadratic couplings that were significant prior to correction remain significant after FDR correction, though some are appropriately downgraded in terms of significance level. Specifically, the T1 externalising female quadratic coupling remains significant at p FDR < .001 (r = .32), and quadratic couplings across dMRI, multimodal, and T1 internalising in females all survive at p FDR < .05. Two intercept–intercept associations also survive FDR correction: T1 externalising in females (r = .08, p FDR = .030) and rs-fMRI externalising in females (r = .09, p FDR = .044). Several borderline associations that were nominally significant prior to correction – including multimodal intercept associations and slope–slope associations – did not survive FDR correction and have been removed from the reported results accordingly.

The revised manuscript updates the Results text, abstract, SI Table 9, and Figure 3 to reflect FDR-corrected p-values throughout, and p-values are now consistently labelled as *p* FDR across the manuscript.

5. The BLGC models don't include any covariates (e.g., SES, race/ethnicity, pubertal status, site), all of which have been shown to impact internalizing/externalizing symptoms. A sensitivity analysis with key covariates would strengthen the manuscript.

Response: We thank Reviewer 2 for this suggestion and agree that demonstrating robustness to key sociodemographic variables is important for the interpretability and credibility of the findings. We address each variable in turn.

Regarding the primary aim of the study, we first wish to clarify that the BLGC framework was selected specifically to characterise the degree to which individual differences in brain maturational trajectories and symptom trajectories co-develop across adolescence – that is, to ask whether the developmental tempo of brain maturation and psychopathology are synchronised at the individual level, above and beyond the normative age-related trends already accounted for by the BAG approach. The goal was not to model all potential sources of variance in brain development or mental health outcomes, but rather to characterise their developmental coupling. These are distinct questions, and including a large set of covariates in the BLGC models would not straightforwardly address the coupling question and could in fact obscure genuine developmental co-variation by partialling out variance that is itself part of the developmental signal.

Regarding SES specifically, we fully appreciate that it is one of the most robust predictors of both brain development and mental health outcomes, and that without sensitivity testing it is reasonable to ask whether the reported associations are driven by SES-related gradients rather than genuine co-development. We have therefore conducted a sensitivity analysis in which a composite baseline measure of socioeconomic status – combining parental and partner income and educational attainment – was included as a time-invariant covariate regressed onto all latent growth factors in each BLGC model. The results were highly consistent with the primary model estimates across all modalities and symptom domains, with negligible attenuation of the quadratic–quadratic coupling estimates that form the core of our findings. This provides direct evidence that the reported BAG–symptom associations are not simply indexing SES-related differences in developmental trajectories. The SES-adjusted coupling estimates are reported in full in SI Table 11, and the sensitivity analysis is described in the methodology and results sections of the revised manuscript.

Lines 339-343: "*To assess robustness of primary coupling estimates to socioeconomic confounding, a sensitivity analysis was conducted including a composite baseline measure of socioeconomic status (SES; combining parental and partner income and educational attainment) as a time-invariant covariate regressed onto all latent growth factors; results are reported in Section 3.2.4 and SI Table 11.*"

Lines 534-541 now read: "*Sensitivity analyses: To assess the robustness of primary coupling estimates to socioeconomic confounding, a sensitivity analysis was conducted in which a composite measure of socioeconomic status (SES; combining parental and partner income and educational attainment at baseline) was included as a time-invariant covariate regressed onto all latent growth factors in each BLGC model. Coupling estimates from the SES-adjusted models were highly consistent with primary model estimates across all modalities and symptom domains, supporting the robustness of the reported associations (SI Table 11).*"

Regarding site, we note that LongComBat harmonisation was applied at the scanner level prior to brain age estimation, removing systematic between-scanner variance from the imaging features used to train the brain age models. This substantially mitigates the need for site as a covariate in the BLGC

models, as the primary source of between-site variance in the brain features has already been addressed at the preprocessing stage.

Regarding race and ethnicity, we made a deliberate and considered decision not to include these as covariates, reflecting growing consensus in the neuroimaging literature that race and ethnicity should not be routinely included as biological covariates without explicit scientific justification, as doing so risks reifying socially constructed categories as biological constructs and can obscure rather than clarify the structural determinants of observed differences (Adkins-Jackson et al., 2022; Cardenas-Iniguez & Robledo Gonzalez, 2024; Flanagin et al., 2021). In the ABCD Study specifically, race and ethnicity are deeply confounded with socioeconomic circumstances, neighbourhood deprivation, and access to resources. We therefore thought it sufficient to include a composite SES measure incorporating household income and parental education as a time-invariant covariate in the sensitivity analysis, capturing the socioeconomic variation most likely to confound BAG–symptom associations. The high consistency between SES-adjusted and primary model estimates provides reassurance that the core findings are robust to this key source of sociodemographic variation, and we believe this represents a more theoretically and ethically grounded approach than routinely including race or ethnicity as a biological control variable.

Regarding pubertal status, comprehensive longitudinal modelling of pubertal effects within the BLGC framework is not straightforward in the present dataset. PDS data in ABCD release 6.0 has incomplete longitudinal coverage across the observation window, meaning that a time-varying pubertal covariate could not be included without substantially reducing the analytic sample or introducing additional missingness into an already sparse four-wave MRI schedule. A baseline-only PDS covariate would not capture the developmental tempo of pubertal change that is most theoretically relevant to the coupling question and would risk conflating pubertal status at a single timepoint with the intercept of the developmental trajectory. Sex was modelled explicitly as a grouping variable throughout, providing partial accounting for the systematic sex differences in pubertal timing that are most relevant here.

We have strengthened the limitations section of the revised manuscript to explicitly acknowledge pubertal timing as an important unmeasured variable in lines 697-706: *"Seventh, although pubertal timing was not included as a covariate in the present analyses, we acknowledge this as a meaningful limitation given the centrality of puberty to adolescent brain maturation and the emergence of internalising psychopathology. The inclusion of sex as a grouping variable provides partial accounting for systematic sex differences in pubertal tempo, and the SES sensitivity analysis offers reassurance that findings are not attributable to a major sociodemographic confound. Nonetheless, pubertal timing, hormonal change, and their interactions with brain maturational trajectories represent important unmeasured factors, and future work incorporating longitudinal pubertal assessments within a developmental coupling framework would be a valuable extension of the present findings."*

6. The effect sizes are mostly small, or small-to-medium at best. Some discussion on what this means in practical or clinical terms would be helpful.

Response: We thank Reviewer 2 for this suggestion. We agree that the modest effect sizes warrant explicit discussion and have added a sentence to the Strengths and Limitations section of the Discussion addressing this point.

The revised manuscript has been updated (lines 706–712), appending the following to the Strengths and Limitations section: *"Finally, the BLGC models capture coordinated change but cannot determine directionality or causal pathways, underscoring the need for future causal designs. The observed effect sizes were small to moderate, and while this is consistent with typical brain–behaviour associations in large community-based neuroimaging studies, the modest magnitudes mean that findings should be interpreted cautiously and require replication in clinically ascertained samples before conclusions about clinical relevance can be drawn."*

7. In the GitHub repository, there's no code at all for the BLGC models, the model selection procedure, or sex-difference tests. Code is also needed here for reproducibility.

Response: We thank Reviewer 2 for raising this. We wish to clarify that the complete analysis code for the BLGC models and sex-difference tests has been publicly available our OSF repository (https://osf.io/dmv2f/overview), which we forgot to link in the main manuscript. We apologise for not making this sufficiently clear. We have updated the manuscript to explicitly direct readers to the OSF repository for the full analysis code, which now has the updated SES sensititivity analyses included as well.

Lines 349-350 of the manuscript: "*All analysis code for the BLGC models is publicly available at https://osf.io/dmv2f/overview*."

8. The repository also references ABCD release 5.1 with two waves, but the paper uses release 6.0 with four waves. The discrepancy should be clarified.

Response: We thank Reviewer 2 for flagging this and apologise for the confusion. The repository linked in the original submission contained brain age prediction code from a related earlier project using ABCD release 5.1 with two imaging waves, which formed the methodological foundation for the brain age models used in the present study. The linked repository was not intended as the replication code for the current manuscript. We have now made the updated four-wave ABCD release 6.0 brain age prediction scripts publicly available in a new dedicated repository at https://github.com/dani-beck/brain-age-prediction-4wave, and the full BLGC analysis code remains available at https://osf.io/dmv2f/overview. We have updated the manuscript to reference both repositories clearly so that readers can access all code relevant to the present study.

Reviewer #3 (Remarks to the Author):

This manuscript leverages multiple time points from the ABCD study to examine how trajectories in brain age gap (BAG) in different imaging modalities (structure, function, DWI, multimodal) relate to internalizing and externalizing symptomology. This paper is generally well-written and theoretically motivated, and fills a clear gap in the literature around longitudinal studies mapping BAG and transdiagnostic symptomology in youth. However, my enthusiasm for this paper is dampened by the lack of specificity in which neural pathways are implicated in these developmental processes. I provide suggestions for improving the manuscript below.

1) Overall, it's unclear what can be gleaned about neural pathways linked to symptomology when hundreds of neural measures are included. In other words, there is a lack of specificity in the findings that make it difficult to interpret beyond the idea that brain maturation rates relate to symptomology, which is interesting but not at all mechanistic. This is especially evident in the discussion, which does not provide any interpretation of the development of specific neural networks, regions, or other neural processes supporting adolescent behavior and psychopathology.

Response: We thank Reviewer 3 for this thoughtful comment. The BAG approach is intentionally multivariate and aggregate – summarising deviation from normative brain maturation across hundreds of features into a single interpretable index that captures overall maturational tempo rather than the contribution of any specific region or network. This aggregation is well-suited to the central question of the present study, which concerns whether the overall pace of brain maturation is coupled with symptom development across adolescence, rather than identifying specific neural circuits underlying these associations. We acknowledge that this means findings cannot speak to mechanism at the level of specific networks or regions, and have made this explicit in the revised manuscript. Feature importance profiles for each modality-specific model are provided in SI Figure 4 as partial information about modality-specific contributors to the BAG index.

The revised manuscript has been updated (lines 680–685), expanding the third limitation to read: *"Third, BAG is a statistical index of multivariate deviation rather than a direct biological marker, and its interpretation should remain cautious. Relatedly, the aggregate nature of the BAG index precludes attribution of findings to specific neural networks, regions, or maturational processes, and mechanistic interpretations at the circuit or network level remain an important direction for future work."*

2) Could the authors clarify why they did not examine additional polynomials beyond quadratic effects, given that there is not a lot of existing literature examining longitudinal BAG, and because they have enough time points to model more complex functions in the data?

Response: We thank Reviewer 3 for raising this important methodological point, and we appreciate the opportunity to clarify our model selection rationale. The reviewer is right that four waves technically permit estimation of higher-order polynomials, and we agree that exploring more complex functional forms is a reasonable consideration given the limited existing literature on longitudinal BAG. In practice, however, we did test the most flexible nonlinear alternative available – latent basis slope models, which freely estimate the shape of change from the data without imposing a pre-specified functional form – and these produced inadmissible solutions across modalities, including Heywood cases and non-positive-definite covariance matrices. This indicated that the available data structure did not provide sufficient information to reliably support freely estimated nonlinear forms, and the centred quadratic model represented the most complex specification that could be estimated stably across modalities. The decision not to pursue higher-order polynomials was therefore empirically motivated rather than a priori. We have documented the full model selection procedure in detail in SI Section 5, including the specifications tested and the criteria used to evaluate and compare them, and we hope this provides the transparency the reviewer is looking for.

3) I understand that the authors have provided variable names for transparency, but for figures like S4, they could provide what the acronym means or label them in a way that is easier for readers to digest.

Response: We thank Reviewer 3 for this suggestion. We agree that the variable name acronyms in SI Figure 4 are not immediately interpretable for readers unfamiliar with the ABCD data structure. Rather than replacing the variable names – which correspond directly to the ABCD data dictionary and SI Tables 3–5 and are retained for transparency and reproducibility – we have expanded the SI Figure 4 caption to describe the top contributing features in plain language for each modality.

The revised supplementary material has been updated with the following addition to the SI Figure 4 caption: *"For reference, the top contributing features in each modality were: T1 – grey/white matter contrast and cortical thickness in frontal and precentral regions, and brainstem volume; dMRI – mean diffusivity in non-occipital white matter tracts; rs-fMRI – connectivity between somatomotor and cingulo-opercular networks; multimodal – mean diffusivity in non-occipital tracts and grey/white matter contrast in precentral cortex."*

4) They report test performance in Table 1, but the paper would be more transparent if it also reported how the training and validation set performed (e.g., MAE, RMSE, R^2, age bias, and residual age correlations).

Response: We thank Reviewer 3 for this suggestion and agree that reporting validation set performance alongside test set metrics improves transparency. We have added validation set performance metrics and age bias information to SI Section 3, where the brain age prediction sample overview already resides, allowing readers to directly compare validation and test set performance and assess model generalisation.

The revised supplementary material has been updated with the following addition to SI Section 3:

"To assess model generalisation, performance metrics were compared between the internal validation set (20% of the training sample) and the hold-out test set. Validation and test set performance were near-identical across all four modalities: T1 (validation: MAE = 1.00, RMSE = 1.27, R^2 = 0.70; test: MAE = 1.00, RMSE = 1.28, R^2 = 0.69), dMRI (validation: MAE = 0.92, RMSE = 1.17, R^2 = 0.74; test: MAE = 0.90, RMSE = 1.15, R^2 = 0.75), rs-fMRI (validation: MAE = 1.36, RMSE = 1.69, R^2 = 0.47; test: MAE = 1.34, RMSE = 1.67, R^2 = 0.47), and multimodal (validation: MAE = 0.86, RMSE = 1.08, R^2 = 0.78; test: MAE = 0.85, RMSE = 1.09, R^2 = 0.78). This indicates that hyperparameter selection was not overly optimistic and that the models generalise well to unseen data. Prior to bias correction, raw BAG values showed the expected negative correlation with chronological age across all modalities ($r = -0.53$ to -0.74), confirming the presence of age bias that was subsequently corrected using the validation set regression procedure described in the Methods. Training set metrics are not reported as they are uninformative for gradient boosting algorithms, which by design fit training data very closely regardless of generalisation performance."

A sentence has also been added to the Brain age prediction section of the main manuscript directing readers to this information (lines 355-356): *"Training and internal validation set performance metrics are reported in SI Section 3 for comparison."*

5) The authors seem to frame neurodevelopment and psychopathology as separate processes in a few places in the intro and discussion (e.g., first sentence of the discussion). The authors may consider rephrasing to acknowledge that neurodevelopment underlies the development of psychopathology, not that they happen to occur in tandem.

Response: We thank Reviewer 3 for this observation and agree that the framing could be improved. We have revised the opening sentence of the Discussion to better reflect the relationship between neurodevelopment and psychopathology, while remaining cautious about strong directional or causal claims that go beyond what the present data can support.

The revised manuscript has been updated (lines 544–546) with: *"Adolescence is a dynamic period of rapid neurobiological change that coincides with the emergence and consolidation of psychopathology[23,27,86,87,] making it a critical window for understanding how individual differences in brain maturation relate to mental health development."*

6) For adolescent neurodevelopment, chronological age alone is often insufficient. Given the authors' claims about developmental timing and sex differences in the discussion, pubertal status, pubertal timing, or hormone related measures should be considered as covariates, moderators, or at least acknowledged as a stronger limitation. They note this in discussion, but this feels central to their argument/interpretation.

Response: We thank Reviewer 3 for this thoughtful and important point. We agree that pubertal timing is central to the developmental framing of the paper, and that given the theoretical claims made in the discussion about sex differences in maturational tempo and the emergence of internalising symptoms, the absence of pubertal measures warrants more than a passing mention. In direct response to the reviewer's suggestion that this should at least be acknowledged as a stronger limitation, we have substantially strengthened the treatment of this issue in the revised manuscript in two ways.

First, we have added a dedicated limitation to the Strengths and Limitations section explicitly and prominently acknowledging pubertal timing as an important unmeasured variable. We explain why comprehensive longitudinal modelling of pubertal effects was not feasible given incomplete wave coverage of PDS data in ABCD release 6.0, and note that a baseline-only covariate would not adequately capture the developmental tempo of pubertal change most relevant to the coupling question. We also note that sex was modelled explicitly as a grouping variable throughout, providing partial accounting for systematic sex differences in pubertal timing. Additionally, as part of the broader revision, we have conducted a new SES sensitivity analysis – including a composite baseline measure of household income and parental education as a time-invariant covariate in all BLGC

models – and results were highly consistent with the primary model estimates across all modalities and symptom domains. This provides reassurance that the core findings are not attributable to a major sociodemographic confound, and offers partial indirect evidence against the concern that unmeasured developmental factors are driving the reported associations.

Second, we wish to clarify that while puberty is theoretically central to adolescent neurodevelopment, the primary aim of the present study was to characterise whether brain maturational trajectories and symptom trajectories co-develop at the individual level – a question the BLGC framework is designed to address – rather than to identify the biological or hormonal mechanisms underlying those trajectories. The discussion references puberty as a plausible contextual factor shaping the developmental window of interest, and we have been careful to frame these interpretations as speculative. Future work incorporating longitudinal pubertal assessments within a developmental coupling framework will be needed to directly test the role of pubertal timing as a moderator of BAG–symptom associations across adolescence.

The revised Strengths and Limitations section now includes the following as a dedicated limitation in lines 697-706: *"Seventh, although pubertal timing was not included as a covariate in the present analyses, we acknowledge this as a meaningful limitation given the centrality of puberty to adolescent brain maturation and the emergence of internalising psychopathology. The inclusion of sex as a grouping variable provides partial accounting for systematic sex differences in pubertal tempo, and the SES sensitivity analysis offers reassurance that findings are not attributable to a major sociodemographic confound. Nonetheless, pubertal timing, hormonal change, and their interactions with brain maturational trajectories represent important unmeasured factors, and future work incorporating longitudinal pubertal assessments within a developmental coupling framework would be a valuable extension of the present findings."*

7) The authors acknowledge the limitations of the BPM in their discussion, but they should more clearly state in the limitations that only symptoms from the prior week were assessed.

Response: We thank Reviewer 3 for this suggestion. The reviewer is correct that the one-week recall window of the BPM is an important constraint that was not explicitly stated in the limitations. We have added this clarification to the existing BPM limitation in the Strengths and Limitations section. The revised manuscript has been updated (lines 689–691) with: *"Fourth, although the BPM provided dense longitudinal symptom data, it is a brief self-report instrument with a one-week recall window and does not capture finer-grained or multi-informant clinical variation."*
