# Supplementary material for: Synchrony between brain age and internalising and externalising symptoms across adolescence
Source: Commun Biol. 2026 Jul 21;9:1509. doi: 10.1038/s42003-026-10722-x (PMC13755446; doi:10.1038/s42003-026-10722-x)
Supplement: Supplementary file 3 [file 42003_2026_10722_MOESM3_ESM.pdf]

Supplementary Table 3. Full parameter estimates from the final multi-group bivariate latent growth curve (BLGC) models. Parameter estimates for all eight BLGC models examining associations between brain-age gap (BAG) trajectories and internalising or externalising symptoms across imaging modalities (T1, dMRI, rs-fMRI, multimodal).

Model	Effect	Est	SE	P value	Est std	Sex
Multi EXT	bag i ~~ bag i	1.351	0.069	< 0.01	1	Female
Multi EXT	bag i ~~ bag i	1.335	0.062	< 0.01	1	Male
Multi INT	bag i ~~ bag i	1.353	0.069	< 0.01	1	Female
Multi INT	bag i ~~ bag i	1.336	0.062	< 0.01	1	Male
T1 EXT	bag i ~~ bag i	2.322	0.093	< 0.01	1	Female
T1 EXT	bag i ~~ bag i	2.11	0.08	< 0.01	1	Male
T1 INT	bag i ~~ bag i	2.333	0.093	< 0.01	1	Female
T1 INT	bag i ~~ bag i	2.108	0.08	< 0.01	1	Male
dMRI EXT	bag i ~~ bag i	1.614	0.062	< 0.01	1	Female
dMRI EXT	bag i ~~ bag i	1.585	0.058	< 0.01	1	Male
dMRI INT	bag i ~~ bag i	1.597	0.077	< 0.01	1	Female
dMRI INT	bag i ~~ bag i	1.599	0.072	< 0.01	1	Male
rsfMRI EXT	bag i ~~ bag i	1.569	0.107	< 0.01	1	Female
rsfMRI EXT	bag i ~~ bag i	1.85	0.112	< 0.01	1	Male
rsfMRI INT	bag i ~~ bag i	1.573	0.107	< 0.01	1	Female
rsfMRI INT	bag i ~~ bag i	1.851	0.112	< 0.01	1	Male
Multi EXT	bag i ~~ bag q	-0.179	0.024	< 0.01	-0.591	Female
Multi EXT	bag i ~~ bag q	-0.195	0.022	< 0.01	-0.595	Male
Multi INT	bag i ~~ bag q	-0.181	0.024	< 0.01	-0.598	Female
Multi INT	bag i ~~ bag q	-0.195	0.022	< 0.01	-0.597	Male
T1 EXT	bag i ~~ bag q	-0.31	0.03	< 0.01	-0.618	Female
T1 EXT	bag i ~~ bag q	-0.237	0.026	< 0.01	-0.624	Male
T1 INT	bag i ~~ bag q	-0.317	0.031	< 0.01	-0.622	Female
T1 INT	bag i ~~ bag q	-0.236	0.026	< 0.01	-0.627	Male
dMRI EXT	bag i ~~ bag q	-0.24	0.019	< 0.01	-0.707	Female
dMRI EXT	bag i ~~ bag q	-0.24	0.019	< 0.01	-0.625	Male
dMRI INT	bag i ~~ bag q	-0.228	0.027	< 0.01	-0.723	Female
dMRI INT	bag i ~~ bag q	-0.249	0.027	< 0.01	-0.624	Male
Multi EXT	bag i ~~ bag s	0.092	0.021	< 0.01	0.2	Female
Multi EXT	bag i ~~ bag s	0.108	0.019	< 0.01	0.263	Male
Multi INT	bag i ~~ bag s	0.09	0.021	< 0.01	0.195	Female
Multi INT	bag i ~~ bag s	0.107	0.019	< 0.01	0.263	Male
T1 EXT	bag i ~~ bag s	0.146	0.027	< 0.01	0.228	Female
T1 EXT	bag i ~~ bag s	0.134	0.024	< 0.01	0.248	Male
T1 INT	bag i ~~ bag s	0.14	0.027	< 0.01	0.22	Female
T1 INT	bag i ~~ bag s	0.134	0.024	< 0.01	0.25	Male
dMRI EXT	bag i ~~ bag s	0.107	0.016	< 0.01	0.228	Female
dMRI EXT	bag i ~~ bag s	0.107	0.016	< 0.01	0.213	Male
dMRI INT	bag i ~~ bag s	0.125	0.024	< 0.01	0.276	Female
dMRI INT	bag i ~~ bag s	0.092	0.023	< 0.01	0.179	Male
rsfMRI EXT	bag i ~~ bag s	0.145	0.062	0.02	0.276	Female
rsfMRI EXT	bag i ~~ bag s	0.199	0.058	< 0.01	0.324	Male
rsfMRI INT	bag i ~~ bag s	0.145	0.063	0.02	0.281	Female
rsfMRI INT	bag i ~~ bag s	0.2	0.059	< 0.01	0.324	Male
Multi EXT	bag i ~~ ext q	-0.009	0.003	< 0.01	-0.139	Female
Multi EXT	bag i ~~ ext q	-0.003	0.003	0.23	-0.059	Male
T1 EXT	bag i ~~ ext q	-0.007	0.003	0.03	-0.085	Female

T1 EXT	bag i ~~ ext q	-0.004	0.003	0.15	-0.06	Male
dMRI EXT	bag i ~~ ext q	-0.004	0.002	0.08	-0.056	Female
dMRI EXT	bag i ~~ ext q	-0.004	0.002	0.08	-0.057	Male
rsfMRI EXT	bag i ~~ ext q	-0.009	0.003	< 0.01	-0.143	Female
rsfMRI EXT	bag i ~~ ext q	0.002	0.003	0.53	0.032	Male
Multi EXT	bag i ~~ ext s	-0.005	0.009	0.56	-0.021	Female
Multi EXT	bag i ~~ ext s	0.012	0.008	0.13	0.053	Male
T1 EXT	bag i ~~ ext s	-0.008	0.01	0.43	-0.025	Female
T1 EXT	bag i ~~ ext s	0.005	0.009	0.63	0.015	Male
dMRI EXT	bag i ~~ ext s	-0.004	0.006	0.52	-0.015	Female
dMRI EXT	bag i ~~ ext s	-0.004	0.006	0.52	-0.016	Male
rsfMRI EXT	bag i ~~ ext s	0.007	0.011	0.51	0.028	Female
rsfMRI EXT	bag i ~~ ext s	-0.002	0.01	0.84	-0.007	Male
Multi INT	bag i ~~ int q	-0.011	0.004	< 0.01	-0.11	Female
Multi INT	bag i ~~ int q	-0.002	0.003	0.57	-0.024	Male
T1 INT	bag i ~~ int q	-0.005	0.004	0.28	-0.039	Female
T1 INT	bag i ~~ int q	0.001	0.003	0.78	0.01	Male
dMRI INT	bag i ~~ int q	-0.008	0.004	0.07	-0.076	Female
dMRI INT	bag i ~~ int q	-0.001	0.003	0.75	-0.014	Male
rsfMRI INT	bag i ~~ int q	-0.007	0.005	0.19	-0.063	Female
rsfMRI INT	bag i ~~ int q	0.002	0.004	0.64	0.022	Male
Multi INT	bag i ~~ int s	-0.012	0.014	0.40	-0.029	Female
Multi INT	bag i ~~ int s	0.016	0.01	0.10	0.053	Male
T1 INT	bag i ~~ int s	-0.002	0.015	0.88	-0.004	Female
T1 INT	bag i ~~ int s	0.014	0.011	0.22	0.038	Male
dMRI INT	bag i ~~ int s	0	0.014	0.99	0	Female
dMRI INT	bag i ~~ int s	-0.003	0.011	0.79	-0.009	Male
rsfMRI INT	bag i ~~ int s	0.005	0.016	0.75	0.013	Female
rsfMRI INT	bag i ~~ int s	0.011	0.014	0.43	0.031	Male
Multi EXT	bag i ~~ out i	0.114	0.053	0.03	0.063	Female
Multi EXT	bag i ~~ out i	0.119	0.05	0.02	0.069	Male
Multi INT	bag i ~~ out i	0.098	0.068	0.15	0.042	Female
Multi INT	bag i ~~ out i	0.099	0.049	0.04	0.058	Male
T1 EXT	bag i ~~ out i	0.18	0.061	< 0.01	0.076	Female
T1 EXT	bag i ~~ out i	0.092	0.057	0.11	0.041	Male
T1 INT	bag i ~~ out i	0.105	0.078	0.18	0.034	Female
T1 INT	bag i ~~ out i	0.02	0.056	0.72	0.009	Male
dMRI EXT	bag i ~~ out i	0.032	0.04	0.42	0.016	Female
dMRI EXT	bag i ~~ out i	0.032	0.04	0.42	0.016	Male
dMRI INT	bag i ~~ out i	0.039	0.074	0.60	0.015	Female
dMRI INT	bag i ~~ out i	-0.024	0.054	0.66	-0.012	Male
rsfMRI EXT	bag i ~~ out i	0.166	0.065	0.01	0.086	Female
rsfMRI EXT	bag i ~~ out i	-0.006	0.062	0.93	-0.003	Male
rsfMRI INT	bag i ~~ out i	0.116	0.083	0.16	0.047	Female
rsfMRI INT	bag i ~~ out i	-0.052	0.064	0.41	-0.025	Male
Multi EXT	bag q ~~ bag q	0.068	0.014	< 0.01	1	Female
Multi EXT	bag q ~~ bag q	0.08	0.014	< 0.01	1	Male
Multi INT	bag q ~~ bag q	0.068	0.014	< 0.01	1	Female
Multi INT	bag q ~~ bag q	0.08	0.014	< 0.01	1	Male
T1 EXT	bag q ~~ bag q	0.108	0.018	< 0.01	1	Female
T1 EXT	bag q ~~ bag q	0.068	0.016	< 0.01	1	Male
T1 INT	bag q ~~ bag q	0.112	0.018	< 0.01	1	Female
T1 INT	bag q ~~ bag q	0.067	0.016	< 0.01	1	Male
dMRI EXT	bag q ~~ bag q	0.071	0.015	< 0.01	1	Female
dMRI EXT	bag q ~~ bag q	0.093	0.014	< 0.01	1	Male

dMRI INT	bag q ~~ bag q	0.062	0.017	< 0.01	1	Female
dMRI INT	bag q ~~ bag q	0.1	0.016	< 0.01	1	Male
Multi EXT	bag q ~~ bag s	0.01	0.02	0.60	0.098	Female
Multi EXT	bag q ~~ bag s	0.01	0.019	0.59	0.101	Male
Multi INT	bag q ~~ bag s	0.011	0.02	0.58	0.105	Female
Multi INT	bag q ~~ bag s	0.009	0.019	0.63	0.092	Male
T1 EXT	bag q ~~ bag s	-0.018	0.026	0.50	-0.128	Female
T1 EXT	bag q ~~ bag s	-0.007	0.024	0.75	-0.077	Male
T1 INT	bag q ~~ bag s	-0.017	0.026	0.52	-0.12	Female
T1 INT	bag q ~~ bag s	-0.008	0.024	0.75	-0.08	Male
dMRI EXT	bag q ~~ bag s	0.009	0.015	0.55	0.094	Female
dMRI EXT	bag q ~~ bag s	0.009	0.015	0.55	0.076	Male
dMRI INT	bag q ~~ bag s	-0.017	0.023	0.45	-0.194	Female
dMRI INT	bag q ~~ bag s	0.031	0.021	0.14	0.237	Male
Multi EXT	bag q ~~ ext i	-0.019	0.021	0.35	-0.048	Female
Multi EXT	bag q ~~ ext i	-0.051	0.02	0.01	-0.121	Male
T1 EXT	bag q ~~ ext i	-0.1	0.023	< 0.01	-0.194	Female
T1 EXT	bag q ~~ ext i	-0.068	0.02	< 0.01	-0.167	Male
dMRI EXT	bag q ~~ ext i	-0.024	0.016	0.13	-0.056	Female
dMRI EXT	bag q ~~ ext i	-0.024	0.016	0.13	-0.049	Male
Multi EXT	bag q ~~ ext s	0.005	0.003	0.15	0.087	Female
Multi EXT	bag q ~~ ext s	0.001	0.003	0.84	0.012	Male
T1 EXT	bag q ~~ ext s	0.006	0.004	0.11	0.082	Female
T1 EXT	bag q ~~ ext s	-0.001	0.003	0.76	-0.019	Male
dMRI EXT	bag q ~~ ext s	0.001	0.003	0.57	0.026	Female
dMRI EXT	bag q ~~ ext s	0.001	0.003	0.57	0.024	Male
Multi INT	bag q ~~ int i	-0.068	0.026	< 0.01	-0.131	Female
Multi INT	bag q ~~ int i	-0.057	0.019	< 0.01	-0.136	Male
T1 INT	bag q ~~ int i	-0.073	0.029	0.01	-0.109	Female
T1 INT	bag q ~~ int i	-0.018	0.02	0.36	-0.045	Male
dMRI INT	bag q ~~ int i	-0.021	0.028	0.46	-0.041	Female
dMRI INT	bag q ~~ int i	-0.014	0.021	0.50	-0.029	Male
Multi INT	bag q ~~ int s	0.003	0.005	0.57	0.034	Female
Multi INT	bag q ~~ int s	0.001	0.004	0.81	0.014	Male
T1 INT	bag q ~~ int s	-0.001	0.005	0.90	-0.007	Female
T1 INT	bag q ~~ int s	-0.001	0.004	0.76	-0.018	Male
dMRI INT	bag q ~~ int s	0.003	0.006	0.55	0.041	Female
dMRI INT	bag q ~~ int s	0	0.004	0.97	-0.002	Male
Multi EXT	bag q ~~ out q	0.003	0.001	< 0.01	0.243	Female
Multi EXT	bag q ~~ out q	0.002	0.001	0.12	0.124	Male
Multi INT	bag q ~~ out q	0.005	0.001	< 0.01	0.228	Female
Multi INT	bag q ~~ out q	0.002	0.001	0.20	0.09	Male
T1 EXT	bag q ~~ out q	0.006	0.001	< 0.01	0.317	Female
T1 EXT	bag q ~~ out q	0.003	0.001	< 0.01	0.236	Male
T1 INT	bag q ~~ out q	0.004	0.002	< 0.01	0.152	Female
T1 INT	bag q ~~ out q	0	0.001	0.98	-0.002	Male
dMRI EXT	bag q ~~ out q	0.002	0.001	< 0.01	0.157	Female
dMRI EXT	bag q ~~ out q	0.002	0.001	< 0.01	0.139	Male
dMRI INT	bag q ~~ out q	0.004	0.002	< 0.01	0.213	Female
dMRI INT	bag q ~~ out q	0	0.001	0.99	-0.001	Male
Multi EXT	bag s ~~ bag s	0.159	0.02	< 0.01	1	Female
Multi EXT	bag s ~~ bag s	0.127	0.019	< 0.01	1	Male
Multi INT	bag s ~~ bag s	0.157	0.02	< 0.01	1	Female
Multi INT	bag s ~~ bag s	0.125	0.019	< 0.01	1	Male
T1 EXT	bag s ~~ bag s	0.176	0.023	< 0.01	1	Female

T1 EXT	bag s ~~ bag s	0.138	0.021	< 0.01	1	Male
T1 INT	bag s ~~ bag s	0.175	0.023	< 0.01	1	Female
T1 INT	bag s ~~ bag s	0.137	0.021	< 0.01	1	Male
dMRI EXT	bag s ~~ bag s	0.137	0.021	< 0.01	1	Female
dMRI EXT	bag s ~~ bag s	0.159	0.02	< 0.01	1	Male
dMRI INT	bag s ~~ bag s	0.128	0.023	< 0.01	1	Female
dMRI INT	bag s ~~ bag s	0.167	0.02	< 0.01	1	Male
rsfMRI EXT	bag s ~~ bag s	0.174	0.073	< 0.01	1	Female
rsfMRI EXT	bag s ~~ bag s	0.205	0.063	< 0.01	1	Male
rsfMRI INT	bag s ~~ bag s	0.168	0.073	0.02	1	Female
rsfMRI INT	bag s ~~ bag s	0.206	0.063	< 0.01	1	Male
Multi EXT	bag s ~~ ext i	-0.013	0.022	0.56	-0.021	Female
Multi EXT	bag s ~~ ext i	-0.005	0.021	0.81	-0.01	Male
T1 EXT	bag s ~~ ext i	-0.058	0.023	0.01	-0.088	Female
T1 EXT	bag s ~~ ext i	-0.056	0.021	< 0.01	-0.096	Male
dMRI EXT	bag s ~~ ext i	-0.021	0.017	0.23	-0.036	Female
dMRI EXT	bag s ~~ ext i	-0.021	0.017	0.23	-0.033	Male
rsfMRI EXT	bag s ~~ ext i	-0.045	0.045	0.32	-0.07	Female
rsfMRI EXT	bag s ~~ ext i	-0.046	0.041	0.26	-0.068	Male
Multi EXT	bag s ~~ ext q	0	0.001	0.81	0.013	Female
Multi EXT	bag s ~~ ext q	0.001	0.001	0.64	0.03	Male
T1 EXT	bag s ~~ ext q	0.002	0.001	0.10	0.089	Female
T1 EXT	bag s ~~ ext q	0.002	0.001	0.12	0.094	Male
dMRI EXT	bag s ~~ ext q	0.001	0.001	0.13	0.072	Female
dMRI EXT	bag s ~~ ext q	0.001	0.001	0.13	0.067	Male
rsfMRI EXT	bag s ~~ ext q	-0.003	0.002	0.25	-0.128	Female
rsfMRI EXT	bag s ~~ ext q	0.002	0.002	0.38	0.093	Male
Multi INT	bag s ~~ int i	-0.041	0.027	0.13	-0.051	Female
Multi INT	bag s ~~ int i	-0.03	0.021	0.16	-0.057	Male
T1 INT	bag s ~~ int i	-0.028	0.028	0.32	-0.033	Female
T1 INT	bag s ~~ int i	-0.048	0.021	0.02	-0.084	Male
dMRI INT	bag s ~~ int i	-0.022	0.03	0.47	-0.029	Female
dMRI INT	bag s ~~ int i	-0.041	0.024	0.09	-0.065	Male
rsfMRI INT	bag s ~~ int i	0.061	0.058	0.30	0.075	Female
rsfMRI INT	bag s ~~ int i	-0.066	0.044	0.13	-0.096	Male
Multi INT	bag s ~~ int q	0.001	0.001	0.36	0.04	Female
Multi INT	bag s ~~ int q	0.001	0.001	0.26	0.07	Male
T1 INT	bag s ~~ int q	0.001	0.002	0.46	0.035	Female
T1 INT	bag s ~~ int q	0.001	0.001	0.32	0.053	Male
dMRI INT	bag s ~~ int q	0.002	0.002	0.24	0.07	Female
dMRI INT	bag s ~~ int q	0.002	0.001	0.24	0.065	Male
rsfMRI INT	bag s ~~ int q	-0.004	0.003	0.29	-0.105	Female
rsfMRI INT	bag s ~~ int q	0.002	0.003	0.49	0.068	Male
Multi EXT	bag s ~~ out s	0.009	0.004	0.02	0.103	Female
Multi EXT	bag s ~~ out s	0.007	0.003	0.03	0.107	Male
Multi INT	bag s ~~ out s	0.007	0.006	0.19	0.054	Female
Multi INT	bag s ~~ out s	0.008	0.005	0.09	0.085	Male
T1 EXT	bag s ~~ out s	0.003	0.004	0.41	0.034	Female
T1 EXT	bag s ~~ out s	0	0.004	0.90	-0.006	Male
T1 INT	bag s ~~ out s	-0.003	0.005	0.60	-0.021	Female
T1 INT	bag s ~~ out s	0.006	0.004	0.12	0.068	Male
dMRI EXT	bag s ~~ out s	0.004	0.003	0.16	0.049	Female
dMRI EXT	bag s ~~ out s	0.004	0.003	0.16	0.048	Male
dMRI INT	bag s ~~ out s	0.006	0.006	0.31	0.054	Female
dMRI INT	bag s ~~ out s	0.008	0.005	0.10	0.072	Male

rsfMRI EXT	bag s ~~ out s	0.011	0.008	0.18	0.125	Female
rsfMRI EXT	bag s ~~ out s	0.011	0.007	0.12	0.116	Male
rsfMRI INT	bag s ~~ out s	0.027	0.011	0.02	0.198	Female
rsfMRI INT	bag s ~~ out s	0.013	0.009	0.153	0.112	Male
Multi EXT	ext i ~~ ext i	2.39	0.102	< 0.01	1	Female
Multi EXT	ext i ~~ ext i	2.24	0.096	< 0.01	1	Male
T1 EXT	ext i ~~ ext i	2.438	0.091	< 0.01	1	Female
T1 EXT	ext i ~~ ext i	2.417	0.091	< 0.01	1	Male
dMRI EXT	ext i ~~ ext i	2.484	0.107	< 0.01	1	Female
dMRI EXT	ext i ~~ ext i	2.521	0.098	< 0.01	1	Male
rsfMRI EXT	ext i ~~ ext i	2.392	0.104	< 0.01	1	Female
rsfMRI EXT	ext i ~~ ext i	2.302	0.084	< 0.01	1	Male
Multi EXT	ext q ~~ ext q	0.003	0	< 0.01	1	Female
Multi EXT	ext q ~~ ext q	0.002	0	< 0.01	1	Male
T1 EXT	ext q ~~ ext q	0.003	0	< 0.01	1	Female
T1 EXT	ext q ~~ ext q	0.003	0	< 0.01	1	Male
dMRI EXT	ext q ~~ ext q	0.003	0	< 0.01	1	Female
dMRI EXT	ext q ~~ ext q	0.003	0	< 0.01	1	Male
rsfMRI EXT	ext q ~~ ext q	0.003	0	< 0.01	1	Female
rsfMRI EXT	ext q ~~ ext q	0.002	0	< 0.01	1	Male
Multi EXT	ext q ~~ ext s	0.003	0.001	< 0.01	0.226	Female
Multi EXT	ext q ~~ ext s	0.001	0.001	0.05	0.13	Male
T1 EXT	ext q ~~ ext s	0.003	0.001	< 0.01	0.272	Female
T1 EXT	ext q ~~ ext s	0.002	0.001	< 0.01	0.173	Male
dMRI EXT	ext q ~~ ext s	0.002	0.001	< 0.01	0.177	Female
dMRI EXT	ext q ~~ ext s	0.002	0.001	< 0.01	0.188	Male
rsfMRI EXT	ext q ~~ ext s	0.003	0.001	< 0.01	0.27	Female
rsfMRI EXT	ext q ~~ ext s	0.002	0.001	< 0.01	0.223	Male
Multi EXT	ext s ~~ ext s	0.048	0.003	< 0.01	1	Female
Multi EXT	ext s ~~ ext s	0.038	0.003	< 0.01	1	Male
T1 EXT	ext s ~~ ext s	0.045	0.003	< 0.01	1	Female
T1 EXT	ext s ~~ ext s	0.044	0.003	< 0.01	1	Male
dMRI EXT	ext s ~~ ext s	0.047	0.003	< 0.01	1	Female
dMRI EXT	ext s ~~ ext s	0.042	0.003	< 0.01	1	Male
rsfMRI EXT	ext s ~~ ext s	0.047	0.003	< 0.01	1	Female
rsfMRI EXT	ext s ~~ ext s	0.041	0.003	< 0.01	1	Male
Multi INT	int i ~~ int i	4.009	0.181	< 0.01	1	Female
Multi INT	int i ~~ int i	2.143	0.119	< 0.01	1	Male
T1 INT	int i ~~ int i	4.005	0.166	< 0.01	1	Female
T1 INT	int i ~~ int i	2.399	0.125	< 0.01	1	Male
dMRI INT	int i ~~ int i	4.205	0.185	< 0.01	1	Female
dMRI INT	int i ~~ int i	2.403	0.132	< 0.01	1	Male
rsfMRI INT	int i ~~ int i	3.873	0.157	< 0.01	1	Female
rsfMRI INT	int i ~~ int i	2.328	0.116	< 0.01	1	Male
Multi INT	int q ~~ int q	0.007	0.001	< 0.01	1	Female
Multi INT	int q ~~ int q	0.004	0	< 0.01	1	Male
T1 INT	int q ~~ int q	0.006	0.001	< 0.01	1	Female
T1 INT	int q ~~ int q	0.004	0	< 0.01	1	Male
dMRI INT	int q ~~ int q	0.006	0.001	< 0.01	1	Female
dMRI INT	int q ~~ int q	0.004	0	< 0.01	1	Male
rsfMRI INT	int q ~~ int q	0.007	0.001	< 0.01	1	Female
rsfMRI INT	int q ~~ int q	0.004	0	< 0.01	1	Male
Multi INT	int q ~~ int s	0.01	0.002	< 0.01	0.327	Female
Multi INT	int q ~~ int s	0.004	0.001	< 0.01	0.263	Male
T1 INT	int q ~~ int s	0.007	0.001	< 0.01	0.271	Female

T1 INT	int q ~~ int s	0.004	0.001	< 0.01	0.252	Male
dMRI INT	int q ~~ int s	0.007	0.001	< 0.01	0.285	Female
dMRI INT	int q ~~ int s	0.003	0.001	< 0.01	0.197	Male
rsfMRI INT	int q ~~ int s	0.008	0.001	< 0.01	0.28	Female
rsfMRI INT	int q ~~ int s	0.003	0.001	< 0.01	0.184	Male
Multi INT	int s ~~ int s	0.121	0.007	< 0.01	1	Female
Multi INT	int s ~~ int s	0.069	0.005	< 0.01	1	Male
T1 INT	int s ~~ int s	0.102	0.006	< 0.01	1	Female
T1 INT	int s ~~ int s	0.064	0.004	< 0.01	1	Male
dMRI INT	int s ~~ int s	0.106	0.006	< 0.01	1	Female
dMRI INT	int s ~~ int s	0.066	0.004	< 0.01	1	Male
rsfMRI INT	int s ~~ int s	0.108	0.006	< 0.01	1	Female
rsfMRI INT	int s ~~ int s	0.065	0.004	< 0.01	1	Male
Multi EXT	out i ~~ out q	-0.04	0.004	< 0.01	-0.478	Female
Multi EXT	out i ~~ out q	-0.03	0.004	< 0.01	-0.415	Male
Multi INT	out i ~~ out q	-0.07	0.008	< 0.01	-0.413	Female
Multi INT	out i ~~ out q	-0.03	0.006	< 0.01	-0.346	Male
T1 EXT	out i ~~ out q	-0.045	0.004	< 0.01	-0.513	Female
T1 EXT	out i ~~ out q	-0.033	0.004	< 0.01	-0.423	Male
T1 INT	out i ~~ out q	-0.073	0.007	< 0.01	-0.459	Female
T1 INT	out i ~~ out q	-0.035	0.005	< 0.01	-0.366	Male
dMRI EXT	out i ~~ out q	-0.037	0.003	< 0.01	-0.457	Female
dMRI EXT	out i ~~ out q	-0.037	0.003	< 0.01	-0.459	Male
dMRI INT	out i ~~ out q	-0.074	0.008	< 0.01	-0.458	Female
dMRI INT	out i ~~ out q	-0.042	0.006	< 0.01	-0.436	Male
rsfMRI EXT	out i ~~ out q	-0.04	0.004	< 0.01	-0.501	Female
rsfMRI EXT	out i ~~ out q	-0.031	0.004	< 0.01	-0.42	Male
rsfMRI INT	out i ~~ out q	-0.078	0.008	< 0.01	-0.472	Female
rsfMRI INT	out i ~~ out q	-0.034	0.005	< 0.01	-0.371	Male
Multi EXT	out i ~~ out s	0.037	0.013	< 0.01	0.11	Female
Multi EXT	out i ~~ out s	-0.004	0.011	0.72	-0.013	Male
Multi INT	out i ~~ out s	0.222	0.023	< 0.01	0.319	Female
Multi INT	out i ~~ out s	0.055	0.016	< 0.01	0.143	Male
T1 EXT	out i ~~ out s	0.026	0.011	0.01	0.078	Female
T1 EXT	out i ~~ out s	-0.023	0.011	0.03	-0.07	Male
T1 INT	out i ~~ out s	0.204	0.02	< 0.01	0.318	Female
T1 INT	out i ~~ out s	0.029	0.014	0.04	0.073	Male
dMRI EXT	out i ~~ out s	0.015	0.009	0.09	0.045	Female
dMRI EXT	out i ~~ out s	0.015	0.009	0.09	0.047	Male
dMRI INT	out i ~~ out s	0.205	0.022	< 0.01	0.306	Female
dMRI INT	out i ~~ out s	0.05	0.016	< 0.01	0.125	Male
rsfMRI EXT	out i ~~ out s	0.023	0.012	0.05	0.069	Female
rsfMRI EXT	out i ~~ out s	-0.015	0.011	0.16	-0.049	Male
rsfMRI INT	out i ~~ out s	0.185	0.02	< 0.01	0.287	Female
rsfMRI INT	out i ~~ out s	0.057	0.015	< 0.01	0.146	Male
